# Supplementary material for: Development and Inter-Laboratory Validation of Diagnostics Panel for Detection of Biothreat Bacteria Based on MOL-PCR Assay
Source: Microorganisms. 2020 Dec 24;9(1):38. doi: 10.3390/microorganisms9010038 (PMC7823616; doi:10.3390/microorganisms9010038)

Supplementary Material

**Development and inter-laboratory validation of diagnostics panel for detection of biothreat agents based on MOL-PCR assay**

**Jelinkova P., Hrdy J., Markova J., Dresler J., Pajer P., Pavlis O., Branich P., Borilova G., Reichelova M., Babak V., Reslova N., and Kralik, P.**

**Figure S1. Biothreat panel validated in University of Veterinary and Pharmaceutical Sciences Brno** – collaborative validation of established biothreat detection panel based on MOL-PCR reaction. A concentration gradient of DNA isolated from biothreat bacteria **(A)** *B. anthracis*, **(B)** *Y. pestis*, **(C)** *F. tularensis*, **(D)** *B. melitensis*, *B. suis* and *B. abortus* was used to validate, verify and to test the robustness of the method. IC confirmed the correct course of the whole reaction. The exclusivity of the detection probes was confirmed by unrelated DNA (*S. aureus*). Panel was evaluated by MFI after subtracting the NTC (H_2_O only) to the appropriate target.


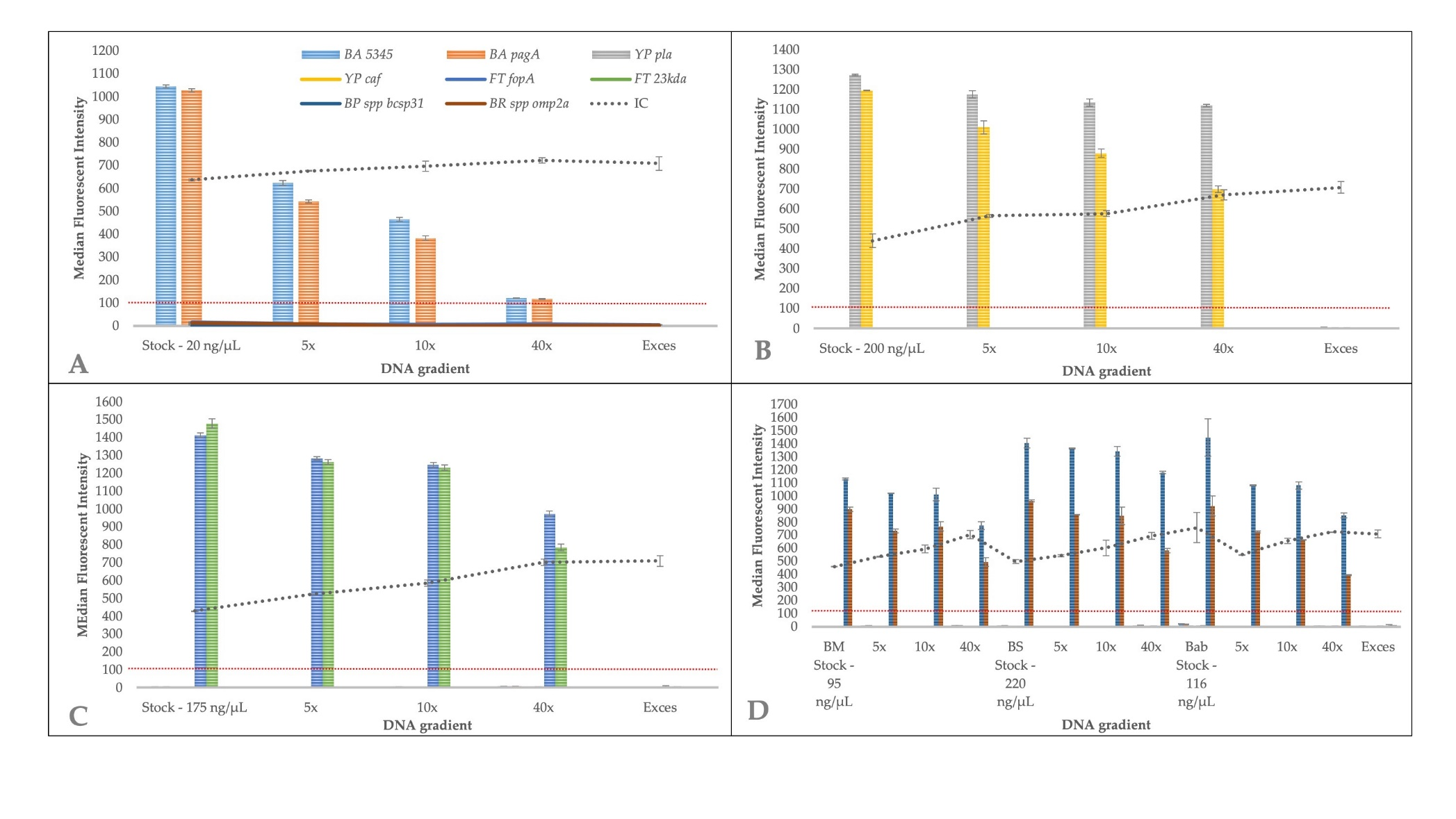


**Figure S2. Biothreat panel validated in Centre of Biological Defence Techonin** – collaborative validation of established biothreat detection panel based on MOL-PCR reaction. A concentration gradient of DNA isolated from biothreat bacteria **(A)** *B. anthracis*, **(B)** *Y. pestis*, **(C)** *F. tularensis*, **(D)** *B. melitensis*, *B. suis* and *B. abortus* was used to validate, verify and test the robustness of the method. IC confirmed the correct course of the whole reaction. The exclusivity of the detection probes was confirmed by unrelated DNA (*S. aureus*). Panel was evaluated by MFI after subtracting the NTC (H_2_O only) to the appropriate target.


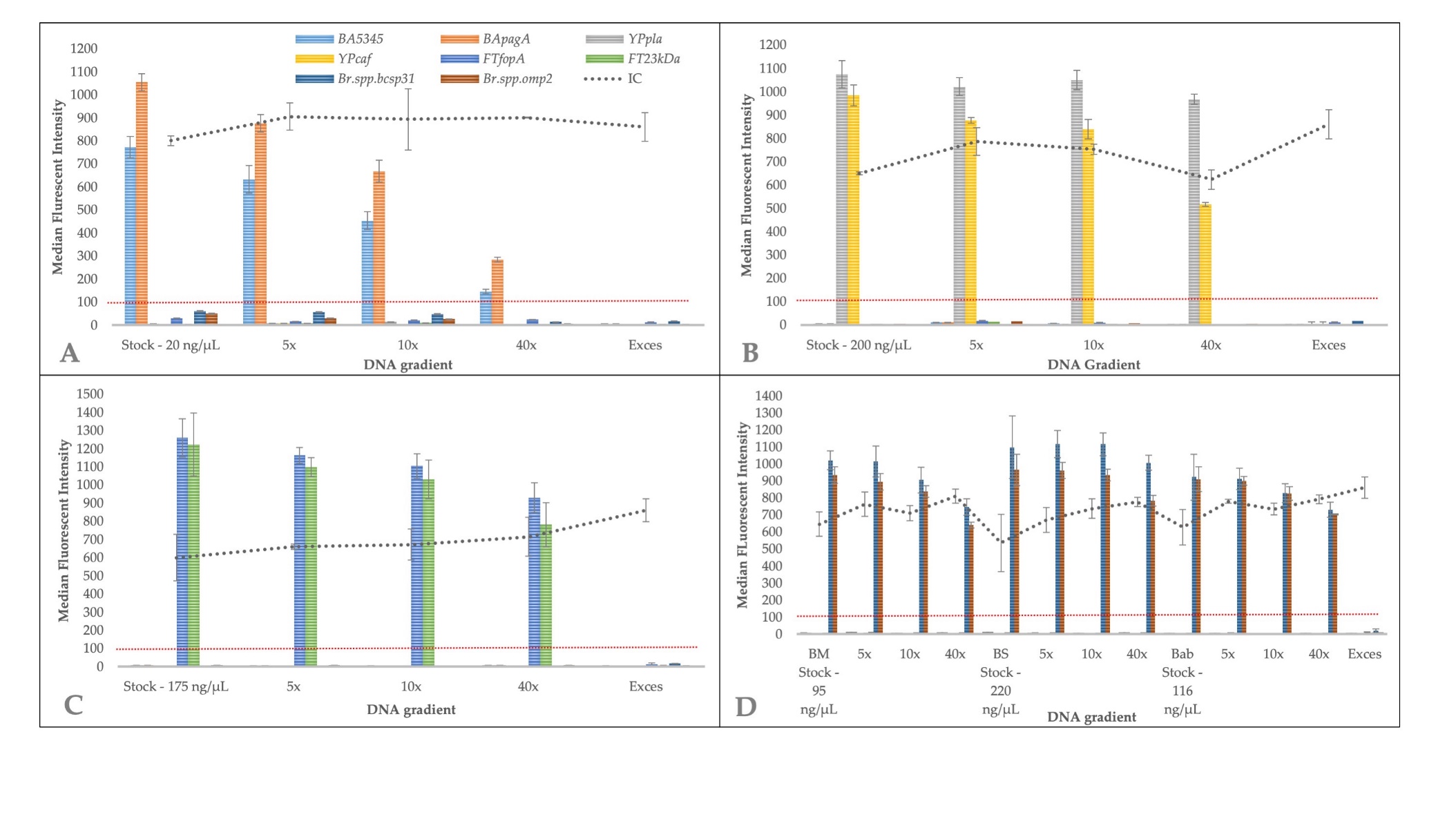


**Figure S3. Biothreat panel validated in Military Veterinary Institute Hlucin** –collaborative validation of established biothreat detection panel based on MOL-PCR reaction. A concentration gradient of DNA isolated from biothreat bacteria **(A)** *B. anthracis*, **(B)** *Y. pestis*, **(C)** *F. tularensis*, **(D)** *B. melitensis*, *B. suis* and *B. abortus* was used to validate, verify and to test the robustness of the method. IC confirmed the correct course of the whole reaction. The exclusivity of the detection probes was confirmed by unrelated DNA (*S. aureus*). Panel was evaluated by the MFI after subtracting the NTC (H_2_O only) from the appropriate target.


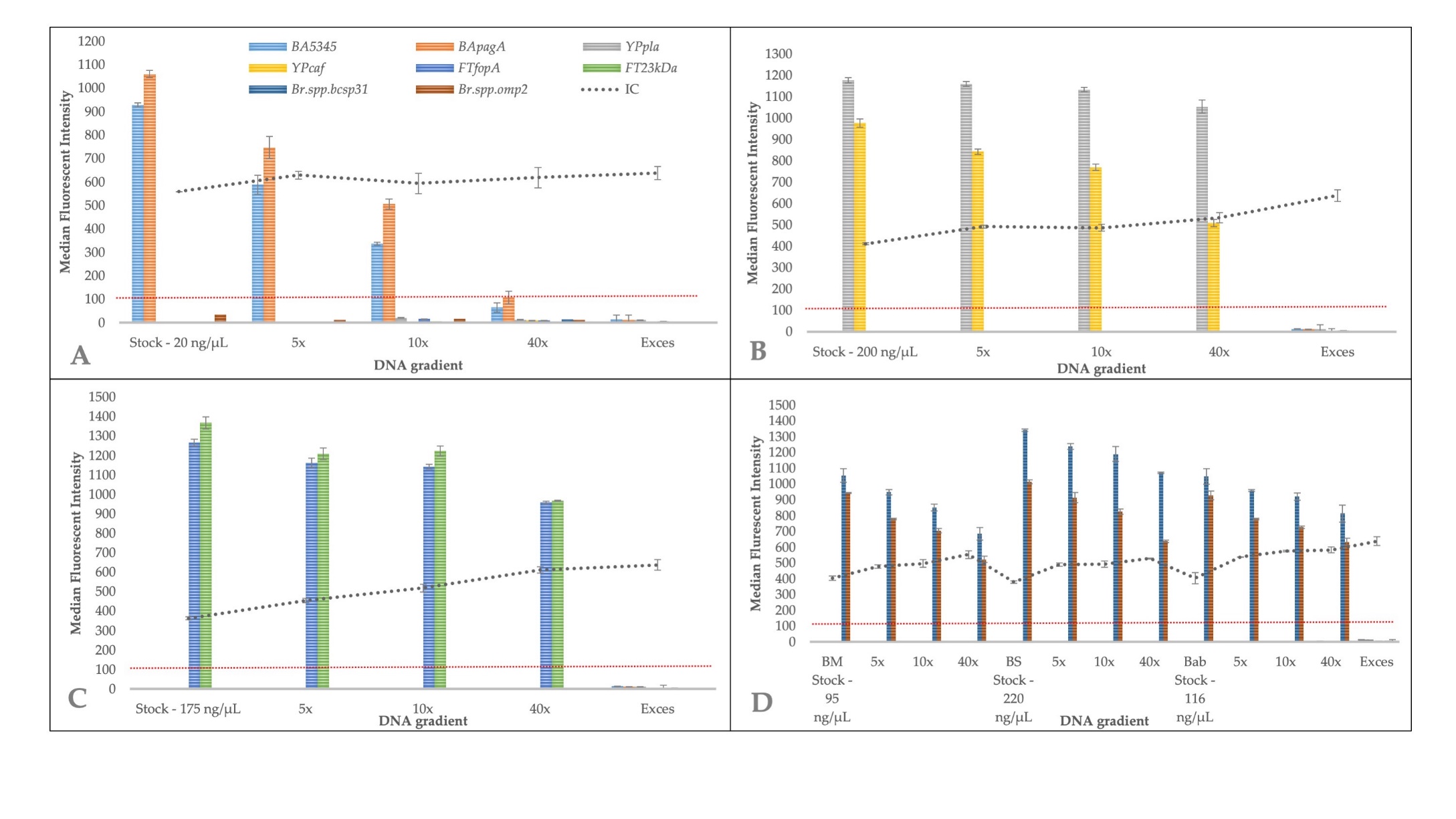

Supplement: Supplementary file 1 [file microorganisms-09-00038-s001.zip › Microorganisms_Supplementary_Material_Jelinkova_et.al._MOL-PCR.docx]
