# Supplementary figures and images for: Development and Inter-Laboratory Validation of Diagnostics Panel for Detection of Biothreat Bacteria Based on MOL-PCR Assay
Source: Microorganisms. 2020 Dec 24;9(1):38. doi: 10.3390/microorganisms9010038 (PMC7823616; doi:10.3390/microorganisms9010038)

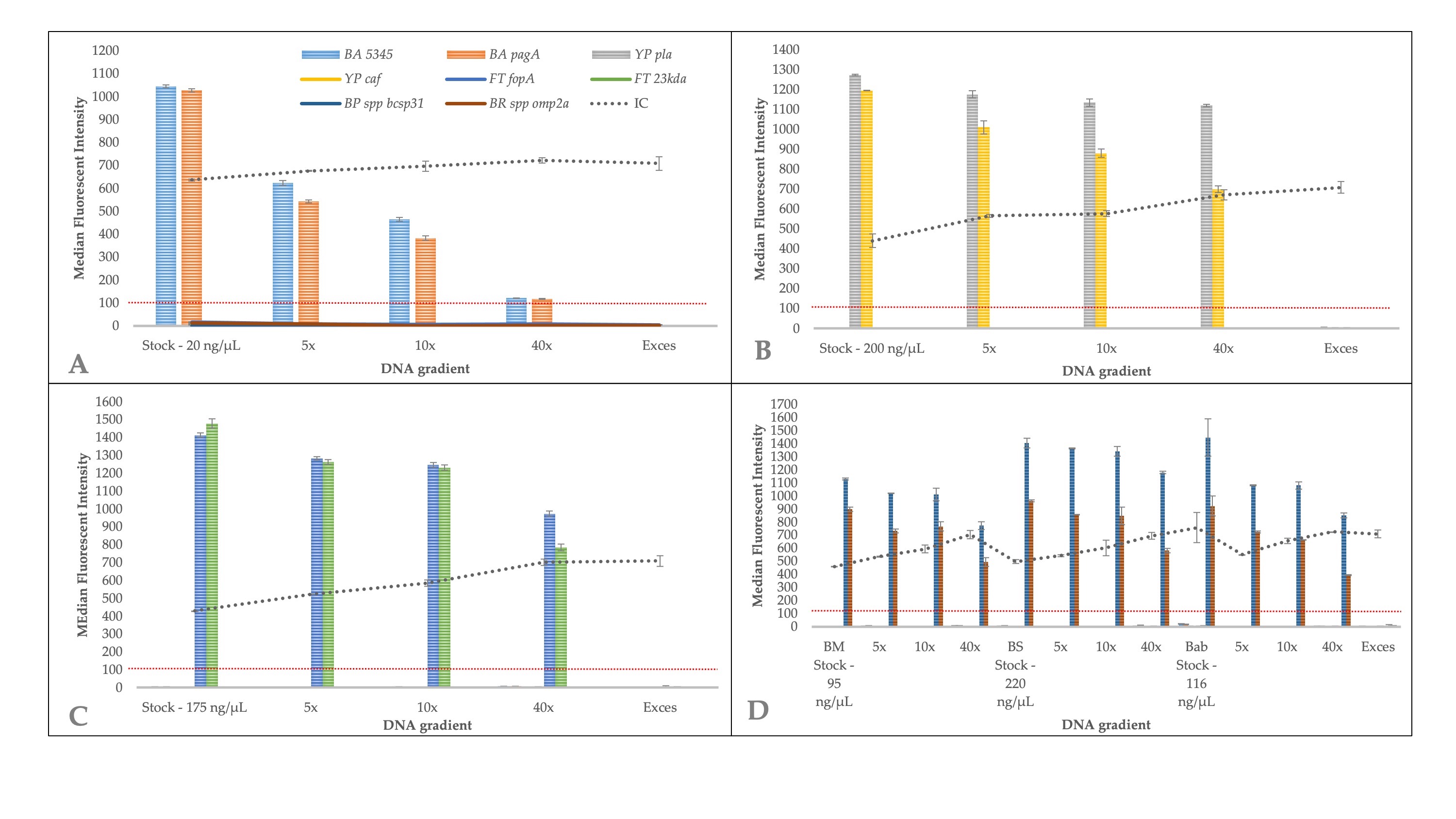

Supplement: Supplementary file 1 [file microorganisms-09-00038-s001.zip › Supplementary_Figure_1.jpg]

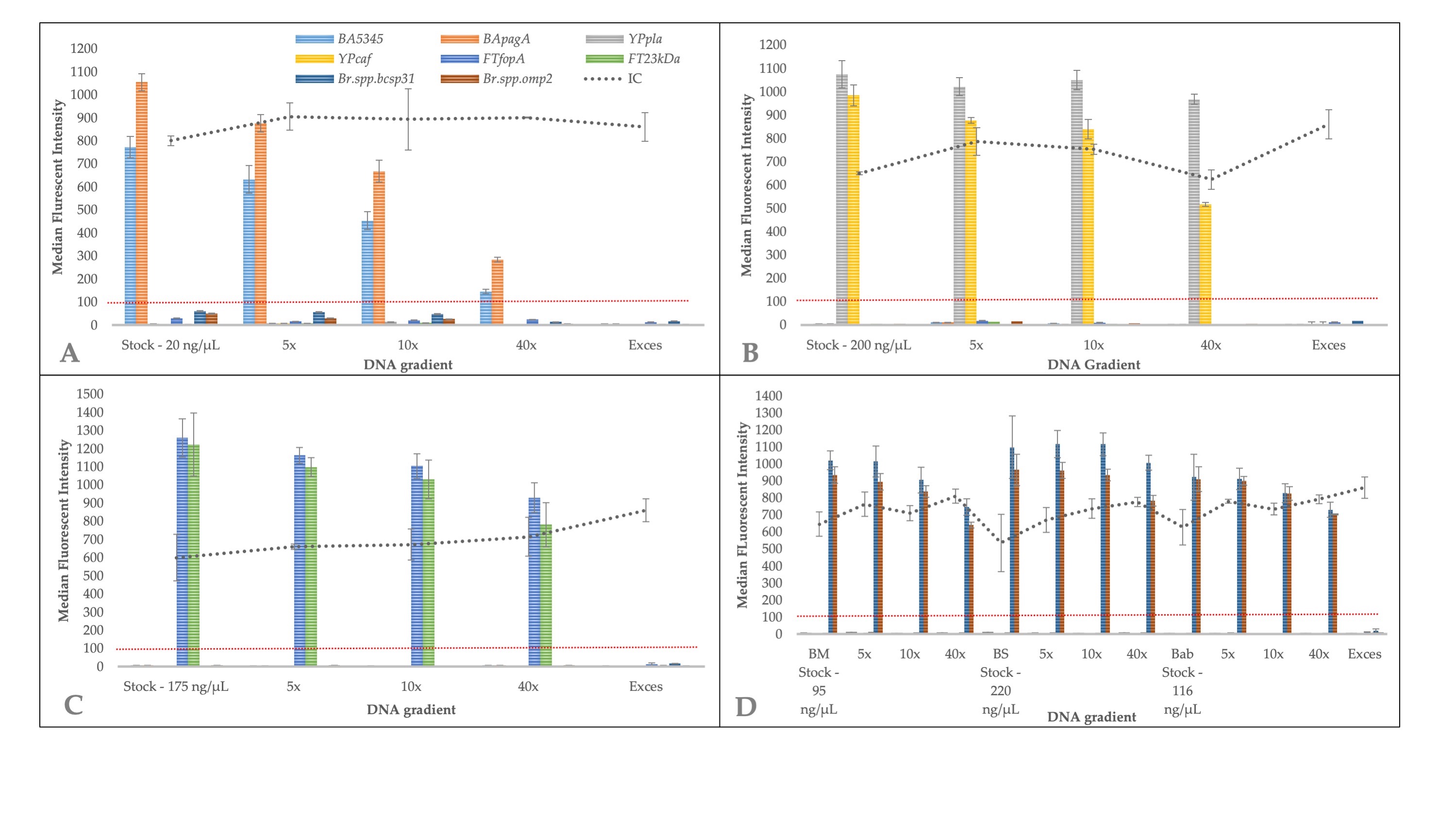

Supplement: Supplementary file 1 [file microorganisms-09-00038-s001.zip › Supplementary_Figure_2.jpg]

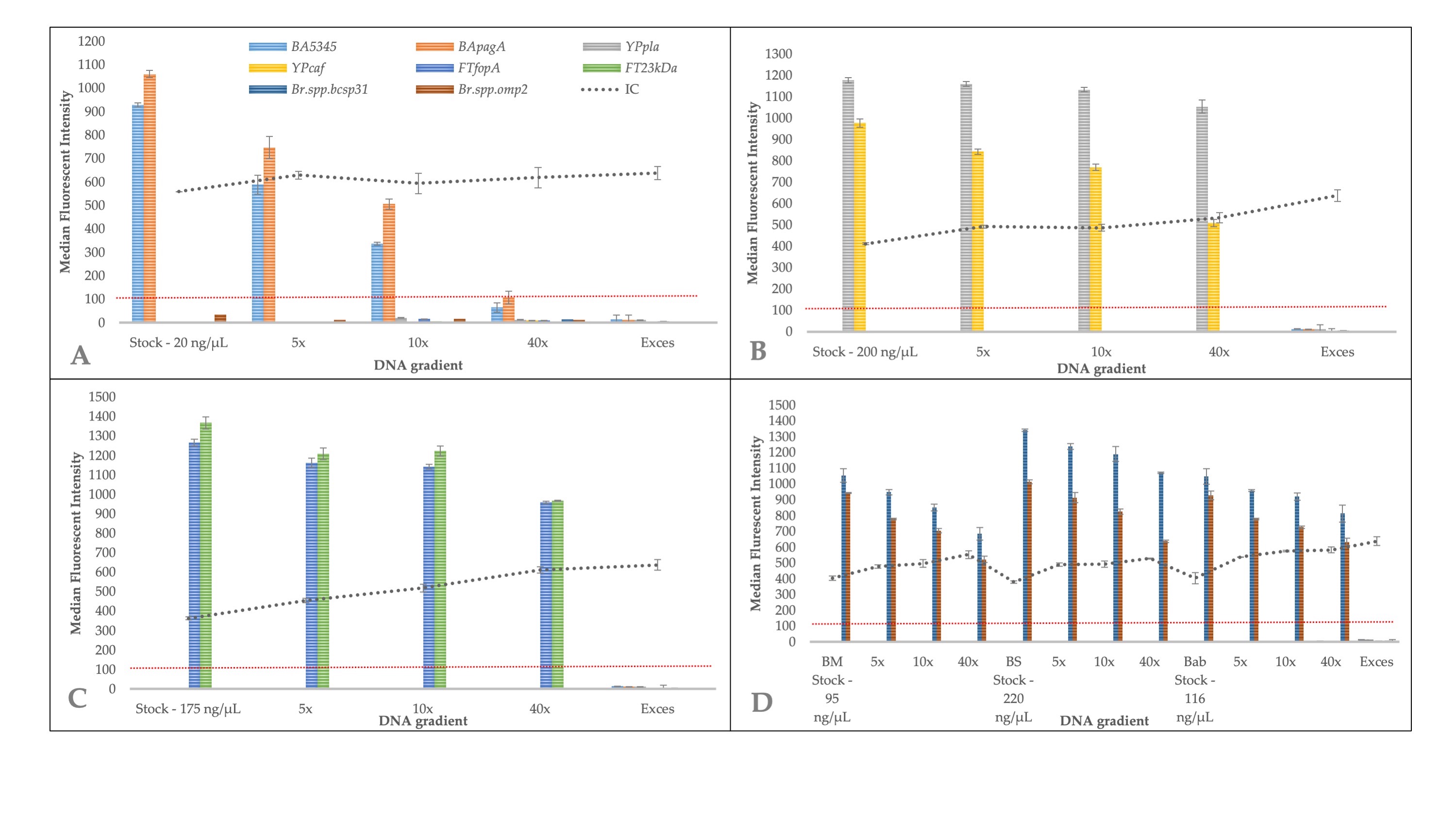

Supplement: Supplementary file 1 [file microorganisms-09-00038-s001.zip › Supplementary_Figure_3.jpg]
